# Supplementary material for: Assessment of the Effects of Triticonazole on Soil and Human Health
Source: Molecules. 2022 Oct 3;27(19):6554. doi: 10.3390/molecules27196554 (PMC9572687; doi:10.3390/molecules27196554)

Figure S1

Illustration of the hydrophilicity of the active site of the alkaline phosphatase from *Bacillus subtilis* (shown as hydrophobicity surface with blue region being hydrophilic and orange regions being hydrophobic) and the C-terminal helix (red cartoon) lying over the active site and controlling the access to this site.

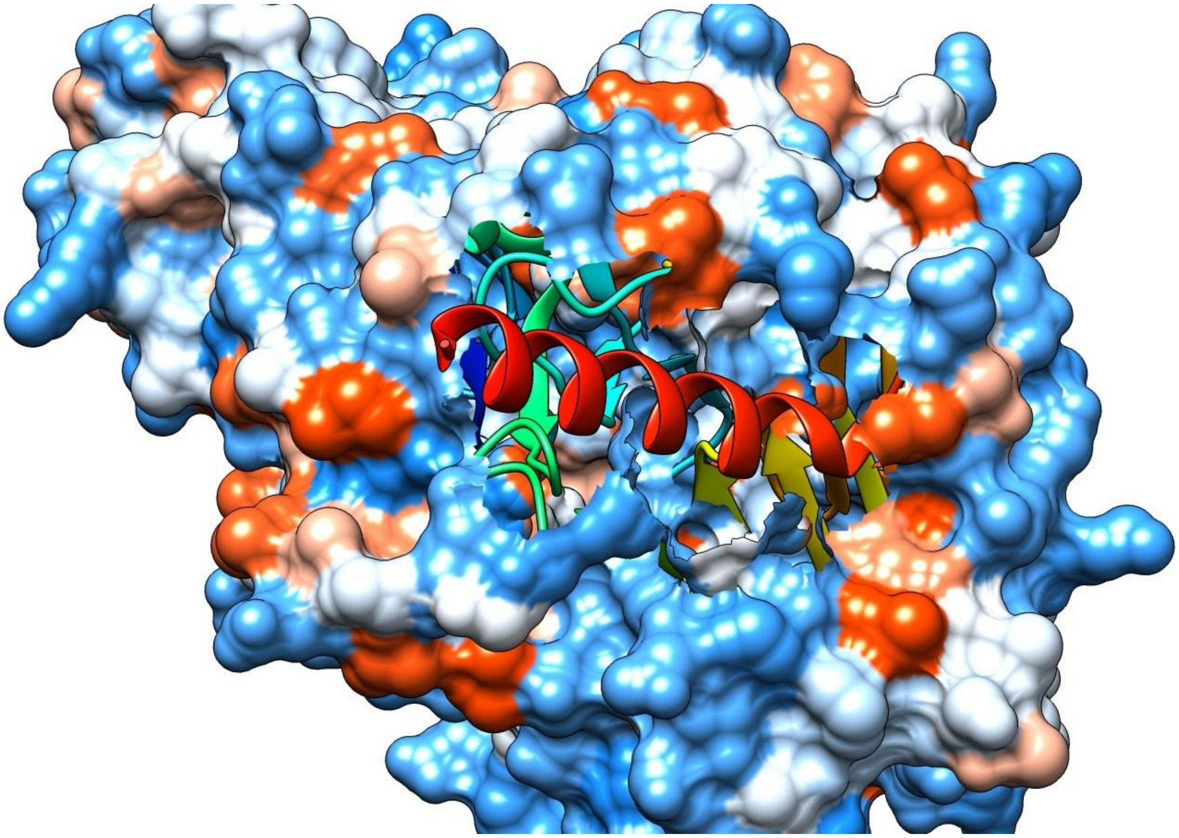

Supplement: Supplementary file 1 [file molecules-27-06554-s001.zip › Figure S1.pdf]
