# Supplementary material for: Assessment of the Effects of Triticonazole on Soil and Human Health
Source: Molecules. 2022 Oct 3;27(19):6554. doi: 10.3390/molecules27196554 (PMC9572687; doi:10.3390/molecules27196554)

Figure S2

Illustration of the hydrophilicity of the active site of the acid phosphatase from *Aspergillus niger*. The protein is shown as hydrophobicity surface with blue region being hydrophilic and orange regions being hydrophobic.

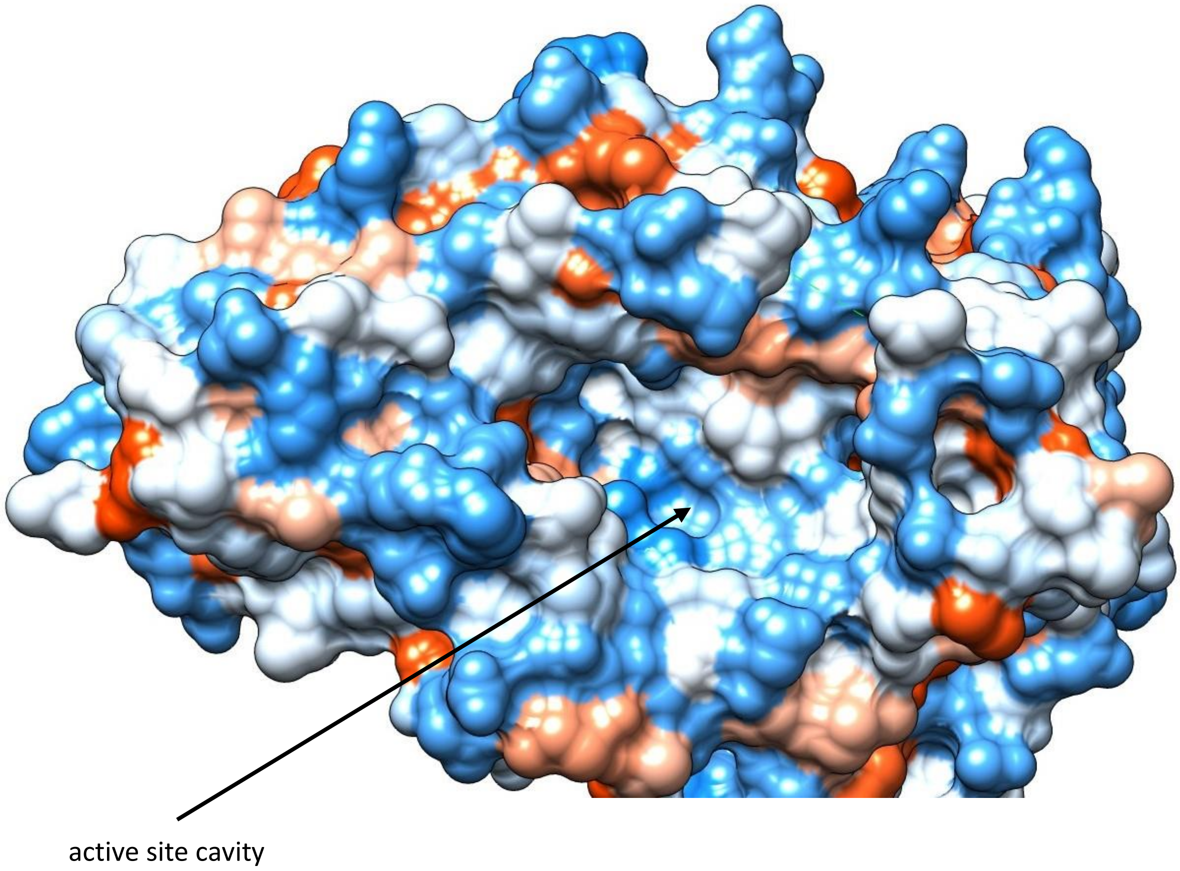

Supplement: Supplementary file 1 [file molecules-27-06554-s001.zip › Figure S2.pdf]
