# Supplementary material for: Assessment of the Effects of Triticonazole on Soil and Human Health
Source: Molecules. 2022 Oct 3;27(19):6554. doi: 10.3390/molecules27196554 (PMC9572687; doi:10.3390/molecules27196554)
Supplement: Supplementary file 1 [file molecules-27-06554-s001.zip › Figure S3.pdf]

Figure S3

(a) Illustration of the hydrophilicity of the active site of the *Bacillus pasteurii* urease. The protein is shown as hydrophobicity surface with blue region being hydrophilic and orange regions being hydrophobic and citrate anion is revealed in yellow sticks.

(b) Illustration of the flexible flap (red cartoon) that changes the active site into a closed conformation around the citrate anion shown in yellow spheres.

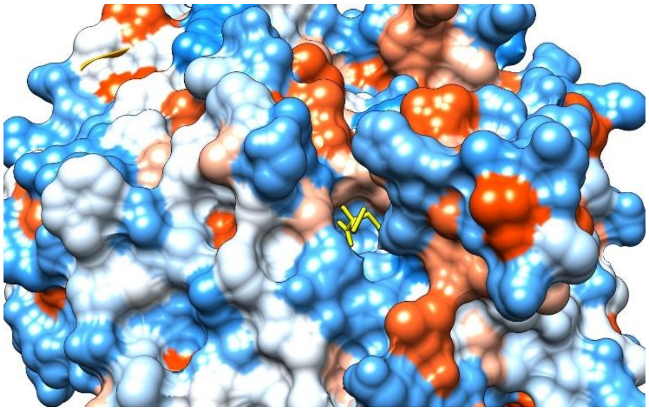

a

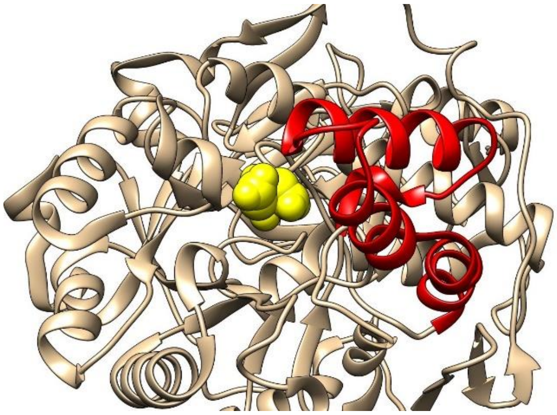

b
