# Supplementary material for: Assessment of the Effects of Triticonazole on Soil and Human Health
Source: Molecules. 2022 Oct 3;27(19):6554. doi: 10.3390/molecules27196554 (PMC9572687; doi:10.3390/molecules27196554)
Supplement: Supplementary file 1 [file molecules-27-06554-s001.zip › molecules-1892735-table S1.pdf]

Table S1. The structural files and the chain that were considered for molecular docking study

Protein	PDB ID	Short characterization of the structural file
soil enzymes		
dehydrogenases	1KEV, chains A, B, C, D	Crystallographic structure of *Clostridium beijerinckii* NADP-dependent alcohol dehydrogenase in complex with dihydro-nicotinamide-adenine-dinucleotide phosphate (NADP) at a resolution of 2.05 Å [48].
	3AUT chains A and B	Crystallographic structure of *Bacillus megaterium* glucose dehydrogenase 4 in complex with 1,4-dihydronicotinamide adenine dinucleotide at a resolution of 2.00 Å [49]. Only the B chain contains the ligand.
	3NT5 chains A and B	Crystallographic structure of *Bacillus subtilis* myo-inositol dehydrogenase in complex with nicotinamide-adenine-dinucleotide and inositol at a resolution of 2.90 Å [50].
	5GTL chains A, B, C, and D	Crystallographic structure of aldehyde dehydrogenase from *Bacillus cereus* in complex with dihydro-nicotinamide-adenine-dinucleotide phosphate at a resolution of 2.90 Å (not published yet).
urease	4AC7 chain C	The crystal structure of *Bacillus pasteurii* urease in complex with the inhibitor citrate at a resolution of 1.50 Å [51]. Only the C chain has been considered as it contains the catalytic domain of urease.
phosphatases	1H2F chain A	Crystallographic structure of the *Bacillus stearothermophilus* phosphatase PhoE in complex with trivanadate at a resolution of 2.00 Å [52].
	1QFX chains A and B	Crystallographic structure of the acid phosphatase from *Aspergillus niger* in complex with 2-acetamido-2-deoxy-beta-D-glucopyranose at a resolution of 2.40 Å [53].
	2YEQ chains A and B	Crystallographic structure of alkaline phosphatase from *Bacillus subtilis* in complex with 3,6,9,12,15,18,21,24-octaoxahexacosan-1-ol at a resolution of 1.98 Å and an active site containing iron and calcium [54].
proteins that are important for assessing the effects of chemicals on the human health		
alpha-1 acid glycoprotein	3KQ0 chain A	Crystallographic structure of human alpha1-acid glycoprotein in complex with (2R)-2,3-dihydroxypropyl acetate at a resolution of 1.80 Å [55].
human serum albumin	4Z69 chains A and I	Crystallographic structure of human serum albumin complexed with palmitic acid and diclofenac at a resolution of 2.19 Å [56].
cytochrome 1A2	2HI4 chain A	Crystallographic structure of human cytochrome 1A2 in complex with alpha-naphthoflavone at a resolution of 1.95 Å [57].
cytochrome 2C9	1OG5 chains A and B	Crystallographic structure of human cytochrome 2C9 in complex with S-warfarin at a resolution of 2.55 Å [58].
cytochrome 2C19	4GQS chains	Crystallographic structure of human cytochrome 2C19 in complex with (4-hydroxy-3,5-dimethylphenyl)(2-methyl-1-benzofuran-3-yl)methanone at a resolution of 2.87 Å [59].

	A, B, C and D	
cytochrome 2D6	4WNV chain A	Crystallographic structure of human cytochrome 2D6 in complex with quinine at a resolution of 2.35 Å [60].
cytochrome 3A4	1W0G chain A	Crystallographic structure of human cytochrome 2D6 in complex with metyrapone at a resolution of 2.35 Å [61].
