# Supplementary material for: Assessment of the Effects of Triticonazole on Soil and Human Health
Source: Molecules. 2022 Oct 3;27(19):6554. doi: 10.3390/molecules27196554 (PMC9572687; doi:10.3390/molecules27196554)
Supplement: Supplementary file 1 [file molecules-27-06554-s001.zip › Table S2-revised.pdf]

Table S2. Noncovalent interactions between stereoisomers of triticonazole (TTZ) and soil enzymes. In parenthesis is given the number of the noncovalent interactions when it is higher than 1. For comparative reasons, this table also contains the noncovalent interactions between the investigated proteins and ligands that are present in their crystallographic structures. NADPH - dihydro-nicotinamide-adenine-dinucleotide phosphate, FBP - 1,6-di-O-phosphono-beta-D-fructofuranose, NAI - 1,4-dihydronicotinamide adenine dinucleotide, ISE- inositol, AMP - adenosine monophosphate.

PDB ID	ligand/ enantiomer	types of nocovalent interactions			
		Hydrophobic interactions	Hydrogen bonds	π stacking	salt bridges
dehydrogenases					
1KEV chain A	NADPH	VAL178	THR38 (2), SER39 (2), ILE175 (2), ALA177, VAL178, GLY179, SER199, ARG200, GLU247, ASN266, TYR267	-	ARG200, LYS340
	(R)-TTZ	VAL178	ARG200 (2), LYS340(2)	-	-
	(S)-TTZ	THR38, VAL178, TYR267	ARG200 (2), LYS340(3)	-	-
1KEV chain B	NADPH	VAL178	ILE175, VAL178, GLY179, SER199(2), ARG200, TYR218, GLY244, TYR267	TYR218 (2)	-
	(R)-TTZ	ARG200	ARG200(2), LEU344	-	-
	(S)-TTZ	THR38, ARG200	ARG200(2)	-	-
1KEV chain C	NADPH	VAL178	THR38(2), SER39(3), ILE175, VAL178, SER199(2), ARG200, GLY244, TYR267	TYR218(2)	ARG200(2), LYS340
	(R)-TTZ	ILE173, VAL178, TYR218(4)	ILE175, SER199(2), ARG200(2)	-	-
	(S)-TTZ	TYR218(3)	SER199(3), ARG200	-	-
1KEV chain D	NADPH	VAL178	THR38(2), SER39(2), HIS42, ILE175, VAL178, SER199, ARG200(2), TYR218, GLY244, GLU247(2), TYR267	TYR218(2)	ARG200, LYS340
	(R)-TTZ	ILE173, VAL197, TYR218	ILE175, GLY176, SER199, ARG200(2)	TYR218	-
	(S)-TTZ	TYR218	SER199(3), ARG200(2)	-	-
3AUT chain A	NAI	PRO188	THR17, LEU19, VAL66, GLY94, TYR158(2), LYS162, MET191(2), THR193, ASN196	-	-
	(R)-TTZ	LEU19, TYR39, ILE195	ASN92, TYR158, LYS162	-	-
	(S)-TTZ	TYR39, ILE195	LEU19, ASN92(2), TYR158, LYS162	-	-

3AUT chain B	NAI	PRO188	THR17(2), LEU19, ASP65, VAL66, GLY94, TYR158(2), LYS162(2), MET191(2), THR193, ASN196	-	-
	(R)-TTZ	LEU19, PRO194, ILE195	-	-	-
	(S)-TTZ	TYR39, VAL95	ASN40	-	-
3NT5 chain A	ISE	-	LYS97, ARG127, HIS155, ASN157, HIS176(2)	-	-
	(R)-TTZ	TYR164, ASP172, TRP272	LYS97, HIS176	-	-
	(S)-TTZ	TYR164, TYR235	LYS97(2)	-	-
3NT5 chain B	ISE	-	LYS97, ARG127, ASN157, HIS176	-	-
	(R)-TTZ	ASP172, TYR235, TRP272(2)	GLY161, TRP272	-	-
	(S)-TTZ	TYR164, TRP272	LYS97	-	-
5GTL chain A	NADPH	PRO166	ASN168, GLU194(2), GLN195, GLY224, SER245(2), THR248(2), GLU266, GYS300, GLN347, GLU397(2)	-	-
	(R)-TTZ	It does not bind to the catalyti site			
	(S)-TTZ	PRO166, TRP167, PHE399(2)	GLU194, GLN195	-	-
5GTL chain B	NADPH	PRO166, TRP167, PHE399(2)	ASN168, GLU194, GLN195, GLY224, GLY244, SER245(3), GLU266, CYS300, GLN347, ARG350, GLU397		LYS191, LYS346
	(R)-TRIT	PRO166, ALA229, PHE242, THR248, TYR251	ILE165, LYS191, GLY224	-	-
	(S)-TRIT	PHR399 (2)	LYS191, GLN195	-	-
5GTL chain C	NADPH	PRO166, TRP167	ASN168, GLU194(2), GLN195, GLY224, SER245(2), THR248(2), TYR251, GLU266, CYS300, GLN347, GLU397, ILE398	PHE399	LYS191, LYS346
	(R)-TRIT	PRO166, TRP167, PHE399	SER245, THR248, LYS346, GLN347, ARG350	-	-
	(S)-TRIT	PRO166, THR248, TYR251	SER245, THR248, LYS346, GLN347	-	-
5GTL chain D	NADPH	PRO166, PHE399	ASN168, GLU194, GLN195, GLY224, SER245(2), THR248, GLU266, GLN347, GLU397 (2)	-	LYS191
	(R)-TRIT	ALA194, PHE242, PHE399	LYS191, GLN195	PHE399	-

	(S)-TRIT	PRO166, PHE399	LYS191 (2), GLU194, GLN195, GLY224	-	-
phosphatase					
1H2F	AMP	ARG9 (2), ASN16, ARG19 (2), ARG20, GLN22 (2), GLY152, MET176	ARG19	-	-
	(R)-TTZ	TRP109, TYR175	ARG19, ARG98	-	-
	(S)-TTZ	ASP101, PRO117	GLN118	-	-
