# Supplementary material for: Assessment of the Effects of Triticonazole on Soil and Human Health
Source: Molecules. 2022 Oct 3;27(19):6554. doi: 10.3390/molecules27196554 (PMC9572687; doi:10.3390/molecules27196554)
Supplement: Supplementary file 1 [file molecules-27-06554-s001.zip › Table S3-revised.pdf]

Table S3. Noncovalent interactions between enatiomers of triticonazole (TTZ) and human cytochromes and plasma proteins, respectively. In parenthesis is given the number of the noncovalent interactions when it is higher than 1. For comparative purposes, this table also contains the noncovalent interactions between the investigated proteins and the ligands that are present in their crystallographic structures: BFH - 2-phenyl-4h-benzo[h]chromen-4-one, OXV - (4-hydroxy-3,5-dimethylphenyl)(2-methyl-1-benzofuran-3-yl)methanone, SWF - S-warfarin, Q19 – quinine, MYT – metyrapone, (2R)-2,3-dihydroxypropyl acetate (JIM) for alpha-1-acid glycoprotein, and diclofenac (DIF) for human serum albumin.

Stereoisomer	Protein and types of interactions			
**HSA CHAIN A**				
	Hydrophobic	Hydrogen bonds	π-staking	Salt Bridges
(R)-TTZ	ARG218, ARG222, LEU238, LEU 260 ILE290, ALA291	ARG275(2)	-	-
(S)-TTZ	LEU260, ILE290, ALA291	ARG218	-	-
DIF 1007	ARG218, LEU219, LEU260	-	-	LYS199, ARG218
**HSA CHAIN I**				
(R)-TTZ	ILE264, ALA291,	ARG218(2)	LYS199	-
(S)-TTZ	TRP2014, LEU219, LEU238(2), LEU260, ALA261, ALA291	LYS199, ARG218, ARG222, ALA291	LYS199	-
DIF 1006	ARG222, PHE223, LEU260	LYS199, ARG218	-	-
**AGP CHAIN A**				
(R)-TTZ	TYR27(2), ARG90, PHE114	GLN66	-	-
(S)-TTZ	TYR27(2), THR47, ILE88, AREG90, ALA99, LEU112, PHE114	GLN66, ARG90	-	-
JIM	ARG90, PHE114	ARG90	-	-
**CYP1A2 CHAIN A**				
(R)-TTZ	THR124, PHE226, ALA317, THR321, LEU382, ILE386, LEU497, THR498	ARG108(2), ILE386, PHE451(2), ARG456	-	-
(S)-TTZ	THR124, PHE226, VAL227, ALA317, ASP320, THR321, LEU382, ILE386(2), LEU497	ARG137, ILE459	-	
BHF	THR118, PHE125, PHE226, PHE260(2), ALA317, ASP320, THR321, LEU382, ILE386, LEU497, THR498	-	PHE226	--
**CYP2C9 CHAIN A**				
(R)-TTZ	LEU208 (2), LEU233, PHE476	-	PHE114	-
(S)-TTZ	PHE100, ALA103, ILE205, LEU208, ILE213, PHE476, ALA477	-	-	-
SWF	ARG97, PHE100(2), VAL113, LEU208, LEU366(2), PRO367, LEU388, PHE476	PHE100, ALA103, ASN217	PHE114, PHE476	
**CYP2C9 CHAIN B**				
(R)-TTZ	ALA103, VAL113, PHE114, LEU208, LEU366	THR364(2)	-	-
(S)-TTZ	ARG97, ILE99, PHE114(2), LEU208, LEU366, PHE476	GLN214, ASN217	-	-
SWF	ARG97, PHE100(3), LEU102, VAL103, LEU366 (2), PRO367, LEU388, PHE476	PHE100, LEU102, ALA103, ASN217	PHE114, PHE476	-

**CYP2C19 CHAIN A**				
(R)-TTZ	VAL113, PHE114, LEU233, ALA297, PHE476	ASN204	-	-
(S)-TTZ	VAL113, PHE114, ILE205, VAL208, ALA297, THR301, LEU366	-	-	-
OXV	VAL113(2), PHE114, ILE205, VAL208, GLU300 (2), THR301, ILE362, LEU366, PHE 476(2), ALA477	-	-	-
**CYP2C19 CHAIN B**				
(R)-TTZ	THR301, LEU361, ILE362(2), LEU366, PHE428	-	-	-
(S)-TTZ	ILE205, GLU300, THR301, LEU366 (2), PHE476 (2)	ARG97, ARG433, ILE434, VAL436	-	-
OXV	VAL113, ILE205, ALA297, GLU300, THR301, LEU366, PHE 476(2), ALA477		-	-
**CYP2C19 CHAIN C**				
(R)-TTZ	VAL113, ALA297, ILE362, LEU366, PHE476	-	-	-
(S)-TTZ	ALA297, LEU366, PHE428	-	-	-
OXV	VAL113, PHE114, ILE205, VAL208, GLU300, THR301, LEU366, PHE476	-	-	-
**CYP2C19 CHAIN D**				
(R)-TTZ	PHE114, ILE205, LEU237, THR301, PHE476(2)	-	-	-
(S)-TTZ	ALA103, PHE114, ILE205, LEU237, THR301, LEU366	ALA297	-	-
OXV	PHE114, ILE205, ALA297, GLU300, THR301, ILE362, LEU366, PHE476 (2), ALA477	-	-	-
**CYP3A4 CHAIN A**				
(R)-TTZ	PHE108, PHE304	ARG105, SER119	-	
(S)-TTZ	ILE301(2), PHE304, ALA305, ILE369, ILE443	-	-	-
MYT	ILE118, PHE137, ILE301(2), PHE302, PHE304, ALA305, THR309, LEU373, PHE435, ILE443	ARG 105(2), ARG130, ARG375, ARG440	-	ARG105(2), ARG130, ARG375, ARG440
